# Picky, hungry eaters in the cold: persistent substrate selectivity among polar pelagic microbial communities

**DOI:** 10.3389/fmicb.2014.00527

**Published:** 2014-10-08

**Authors:** Andrew D. Steen, Carol Arnosti

**Affiliations:** ^1^Department of Marine Sciences, University of North Carolina at Chapel HillChapel Hill, NC, USA; ^2^Department of Earth and Planetary Sciences, University of TennesseeKnoxville, TN, USA

**Keywords:** microbial loop, heterotrophic bacteria, Arctic Ocean, extracellular enzymes, latitudinal gradient

## Abstract

Polar pelagic microbial communities access a narrower range of polysaccharide substrates than communities at lower latitudes. For example, the glucose-containing polysaccharide pullulan is typically not hydrolyzed in fjord waters of Svalbard, even though pullulan is rapidly hydrolyzed in sediments from Svalbard fjords, other polysaccharides are hydrolyzed rapidly in Svalbard waters, and pullulan is hydrolyzed rapidly in temperate waters. The purpose of this study was to investigate potential factors preventing hydrolysis of pullulan in Svalbard fjord waters. To this end, in two separate years, water from Isfjorden, Svalbard, was amended with different carbon sources and/or additional nutrients in order to determine whether increasing the concentration of these potentially-limiting factors would lead to measurable enzymatic activity. Addition of nitrate, phosphate, glucose, or amino acids did not yield detectable pullulan hydrolysis. The only treatment that led to detectable pullulan hydrolysis was extended incubation after the addition of maltotriose (a subunit of pullulan, and potential inducer of pullulanase). In these fjords, the ability to enzymatically access pullulan is likely confined to numerically minor members of the pelagic microbial community. These results are consistent with the hypothesis that pelagic microbial communities at high latitudes exhibit streamlined functionality, focused on a narrower range of substrates, than their temperate counterparts.

## INTRODUCTION

Surface ocean microbial communities differ systematically in their abilities to enzymatically hydrolyze high molecular weight organic matter, and thus to initiate remineralization of high molecular weight substrates (Arnosti et al., 2011). Pelagic microbial communities at high latitude are capable of hydrolyzing a narrower spectrum of soluble substrates than their temperate counterparts, although the underlying reasons for this substrate selectivity are unknown. This narrowing of microbial community function at high latitude parallels decreases in microbial diversity and/or species richness at high latitude (Baldwin et al., 2005; Pommier et al., 2007; Fuhrman et al., 2008; Wietz et al., 2010). Nevertheless, Arctic pelagic heterotrophic microbial communities are active players in the marine carbon cycle, transforming, and respiring a wide range of substrates, despite permanently cold temperatures (Wheeler et al., 1996; Kirchman et al., 2009a). Substrate consumption in Arctic microbial communities is affected by a wide range of factors, including season, location, and nutrient levels (Kirchman et al., 2009b; Nikrad et al., 2012). The functional difference in enzyme activities observed over latitudinal gradients, and the narrow spectrum of substrates hydrolyzed by Arctic microbial communities, may thus be driven by a lack of a nutrient or co-factor required to induce production of specific enzymes by organisms possessing them, or conversely by a lack of organisms with the genes to synthesize the enzymes required to hydrolyze specific substrates.

The supply of labile DOM has been suggested to play a key role in controlling bacterial growth at high latitude (e.g., Kirchman et al., 2009b). Substrate availability in ocean waters is also highly variable: transient events, such as phytoplankton blooms or physical mixing can rapidly change the set of organic substrates present, requiring changes in expression of genes related to organic matter metabolism (Teeling et al., 2012). Here, we investigated whether an expansion in enzymatic capabilities of pelagic microbial communities in waters of the Svalbard archipelago could be achieved through addition of a factor potentially lacking in the water column: mineral nutrients (nitrate and/or phosphate), labile organic carbon or nitrogen, or a specific inducer that might be expected to activate enzyme production. As a test case, we focused on trying to enhance the activity of enzymes that hydrolyze pullulan, a soluble, glucose-containing polysaccharide. Pullulan [α(1,6)-linked maltotriose; maltotriose is α(1,4)-linked glucose], was selected as a test case because in repeated investigations over the past decade, we have not detected pullulanase activity in the water column of the fjords of Svalbard, although it is readily hydrolyzed at a number of sites in temperate waters. Its basic constituent, glucose, is also is found in laminarin, a substrate readily hydrolyzed in Svalbard waters (Arnosti et al., 2005, 2011; Arnosti, 2008; Teske et al., 2011). The lack of hydrolysis of pullulan in the water column at Svalbard is particularly intriguing because pullulanase activity in underlying sediments is high (Arnosti, 2008; Teske et al., 2011), and bacterial isolates have been obtained from Svalbard sea-ice using pullulan as a growth substrate (Groudieva et al., 2004).

## MATERIALS AND METHODS

### SEAWATER COLLECTION

Surface seawater for the pullulanase induction experiments was obtained from Isfjord, Svalb ard (78° 16.55′ *N*, 15° 10.1′ E) on 29 June 2005, and on 3 August 2006. Samples were kept on ice in the dark during transit (ca. 48 h) to the shore-based lab in Ny Ålesund, Svalbard. In order to assess hydrolysis rates of a broad range of polysaccharides, surface, and bottom seawater was also collected from Station J, Smeerenburgfjord (79° 42.8′ *N*, 011° 05.2′E) in 2006 and transported back to Ny Ålesund. Previous work in Svalbard indicates that pelagic extracellular enzyme activities are stable over a timescale of several days (Steen and Arnosti, 2011).

### PULLULANASE INDUCTION EXPERIMENTS

In order to investigate whether addition of carbon or nutrient compounds potentially influenced pullulanase expression, samples were “pre-incubated” with different carbon and/or nutrient compounds prior to addition of fluorescently labeled (fl)-pullulan. In 2005, pre-incubation periods were 24 h and 5 days. In 2006, due to logistical constraints, pre-incubation periods were shortened to 12 h and 3 days. After the pre-incubation period, fl-pullulan or fl-xylan was added to each treatment, and polysaccharide hydrolysis rates were measured as described below. The experimental design in each year is summarized in **Table 1**.

**Table 1:** Hydrolysis rates for xylan and pullulan under different experimental treatments, nmol L^-1^ h^-1^.

Year	Pre-incubation period	Xylan	Pullulan		
2005		No amendment	No amendment	+glucose	+maltotriose	+$NO_{3}^{-}$	+$PO_{4}^{3-}$	+$NO_{3}^{-}$+$PO_{4}^{3-}$
	24 h	4.3	0	0	0	0	0	0
	5 days	6.8	0	0	0.86 ± 0.13	0	0	0
2006			No amendment	+glucose	+maltotriose	+pullulan	+AA	+$NO_{3}^{-}$+$PO_{4}^{3-}$
	12 h	n.m.	0	0	0	0	0	0
	3 days	n.m.	0	0	0	0	0	0

*Glucose, maltotriose, and unlabeled pullulan concentrations were 0.875 μmol L^-1^ monomer-equivalent. $NO_{3}^{-}$ concentration was 40 μM as K$NO_{3}^{-}$; $PO_{4}^{3-}$ concentration was 16 μM as Na_2_HPO_4_. +AA treatment included all 20 genetically-encoded amino acids at equimolar concentrations, for a total concentration of 5.25 μM-C. n.m. indicates that xylan hydrolysis in 2006 was not measured in Isfjorden waters.*

In 2005, experimental treatments consisted of additions of glucose (potentially a labile source of organic carbon; 0.875 μM-C), maltotriose (a trimer of glucose and a constituent of pullulan; 0.875 μM-C), nitrate (potentially a limiting nutrient; 40 μM), phosphate (potentially a limiting nutrient; 16 μM), and nitrate plus phosphate (potentially limiting nutrients; 40 and 16 μM, respectively). Maltotriose and glucose were selected because they have been observed to induce and repress pullulanase activity, respectively (Hope and Dean, 1974; Antranikian, 1990; Nair et al., 2007). Nutrient amendments were included because low nutrient conditions can lead to low pullulanase activity even in the presence of pullulanase inducers (Nair et al., 2007). Additionally, a treatment with no addition was included as a control, and a treatment with no addition, but in which hydrolysis of fl-xylan rather than fl-pullulan was measured, was included as a positive control for microbial hydrolysis of polysaccharides. Xylan was chosen as the positive control since previous work on Svalbard had repeatedly demonstrated rapid hydrolysis of this polysaccharide (e.g., Arnosti et al., 2011). In 2006, experimental treatments consisted of additions of glucose (0.875 μM-C), maltotriose (0.875 μM-C), unlabeled pullulan (0.875 μM-C), an equimolar mix of the 20 genetically-encoded amino acids (as sources of labile carbon and nitrogen; total concentration 5.25 μM-C) and nitrate plus phosphate (40 and 16 μM, respectively). These nutrient concentrations were likely high compared to *in situ* nutrient concentrations, which were previously been observed in the range of 0–10 μM $NH_{4}^{+}$ + $NO_{3}^{-}$ and 0.1–0.7 μM $PO_{4}^{3-}$ in Svalbard waters (Owrid et al., 2000; Iversen and Seuthe, 2011).

### PROTOCOL FOR MEASURING POLYSACCHARIDE HYDROLYSIS RATES

Fl-polysaccharides (pullulan, xylan, arabinogalactan, chondroitin sulfate, fucoidan, and laminarin,) were synthesized as described by Arnosti (Arnosti, 1996, 2003). For each hydrolysis rate measurement, polysaccharides were added to a 50 ml sample, which was divided into three biological replicates. Samples were then incubated at 7 °C (2005) or 4 °C (2006). Subsamples were taken after 3, 8, and 21 days (2005 Isfjorden samples); 3, 7, and 14 days (2006 Isfjorden samples) or 2, 4, 6, 10, and 16 days of incubation (2006 Smeerenburgfjorden samples). Fl-polysaccharide size distributions and hydrolysis rates were calculated as described previously (Arnosti, 1996, 2003).

### HYDROLYSIS OF A BROAD RANGE OF POLYSACCHARIDES IN SMEERENBURGFJORDEN

In 2006, hydrolysis rates of arabinogalactan, chondroitin sulfate, fucoidan, laminarin, pullulan, and xylan were assessed in surface (1 m depth) and bottom (ca. 200 m depth) at station, J, Smeerenburgfjorden. Polysaccharide hydrolysis rates were measured with no pre-incubation period, and without amendments of substrates other than the fl-polysaccharides, as described above.

## RESULTS

**FIGURE 1:**
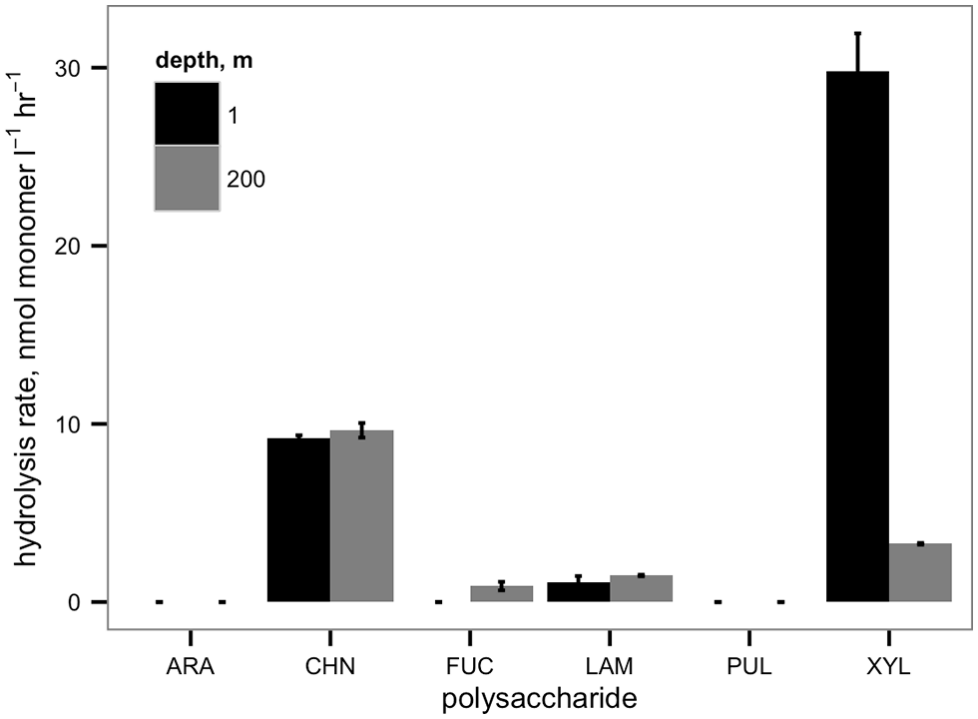
**Hydrolysis rates of six polysaccharides in surface and bottom waters at Station J, Smeerenburgfjord, August 2006.** Bars represent SD. of triplicate incubations. Ara, arabinogalactan; chn, chondroitin sulfate; fuc, fucoidan; lam, laminarin; pul, pullulan; xyl, xylan.

Heterotrophs capable of hydrolyzing polysaccharides were active in the pullulan induction experiments, as shown by maximum xylan hydrolysis rates (measured in a single replicate after 21 days incubation) of 4.3 nM L^-1^ h^-1^ in the 24 h pre-incubation no-addition samples and 6.8 nM L^-1^ h^-1^ in the 5-day pre-incubation no-addition samples (**Table 1**). Moreover, four distinct polysaccharide substrates were hydrolyzed in surface and bottom waters from Station J in Smeerenburgfjord (**Figure 1**), consistent with previous results that pelagic microbial communities in Svalbard are readily capable of accessing specific polysaccharides (Arnosti, 2008; Teske et al., 2011).

Efforts to stimulate use of pullulan as a substrate, however, showed that pullulan is microbially-inaccessible for most of the pelagic community (**Table 1**). Only a single set of treatments—the 5 days pre-incubation with maltotriose—led to hydrolysis of fl-pullulan during the subsequent incubation. Even in this case, substantial incubation time (21 days) was required before detectable hydrolysis of pullulan occurred. Addition of glucose, pullulan, nitrate, phosphate, nitrate, and phosphate, and free amino acids did not lead to measurable pullulanase activity, nor did addition of maltotriose on timescales shorter than the 5 days pre-incubation + 21 days incubation (**Table 1**).

## DISCUSSION

The observation that addition of maltotriose led to measurable pullulanase activity after sufficient pre-incubation is consistent with biochemical control of pullulanase expression, since pullulan is composed of repeating units of maltotriose. Many extracellular polysaccharide hydrolases are regulated by a scheme in which the hydrolase is expressed constitutively at a low level (Béguin and Aubert, 1994). Oligomers of the polysaccharide induce further expression of the hydrolase, while monomers of the polysaccharide (glucose, in this example) repress expression of the homologous hydrolase. This regulation mechanism has been demonstrated for thermophilic bacteria that hydrolyze pullulan (Antranikian, 1990); the current data suggest that pullulanase activity in Svalbard waters may be regulated in a similar manner. The fact that hydrolysis was only detected after 21 days in the +maltotriose, 5-days pre-incubation treatment from 2005 (and was not detectable with shorter pre-incubation periods with either maltotriose or pullulan) further suggests that the microorganisms in fjord waters capable of hydrolyzing pullulan were likely slow-growing as well as initially rare.

Induction of enzymatic pathways can occur on timescales of hours or faster in the lab (Madigan et al., 2000); and less than 2 days for hydrolysis patterns consistent with pullulanase induction in seawater at lower latitudes (Steen et al., 2012). Substrates for which the corresponding extracellular enzyme must be induced may therefore be hydrolyzed quite rapidly, if the final activity of the induced enzyme is high. The combined requirements of growth of a relatively rare member of the microbial community and induction of enzymes, however, could lead to the pattern observed in this study. Slow growth of bacteria in Svalbard waters is consistent with the results of Vadstein (2011), who found no increase in cell numbers over 22 h bioassay experiments in which carbon and nutrients were added to Svalbard fjord water. It is also consistent with results of very long-term incubations of surface water from Station J, in which hydrolysis of *Isochrysis* extract was first observed only after 70 days’ incubation, activity also attributable to initially rare and slow-growing bacteria (Arnosti, 2008).

In many respects, the general absence of pullulanase activity in Svalbard waters is quite surprising, given that it is a soluble, linear polysaccharide composed of glucose. Furthermore, pullulanase functions as a debranching enzyme of starch (Ball and Morell, 2003), a common algal energy storage polysaccharide (Painter, 1983). Hydrolysis of pullulan in Svalbard sediments is rapid (Arnosti, 2008; Teske et al., 2011), and a diverse range of bacterial isolated from sea ice in Svalbard’s fjords can hydrolyze pullulan (Groudieva et al., 2004). In addition, *Aureobasidium pullulans*, a fungus which produces pullulan, has been isolated from Svalbard fjord water (Zalar et al., 2008), so there is very likely a source of pullulan to the water we studied. Furthermore, pelagic communities in Svalbard’s fjords can hydrolyze a number of other polysaccharide substrates, as demonstrated by the incubations carried out with water from Station J (**Figure 1**). The observation that pullulan and arabinogalactan were not hydrolyzed over the time course of incubation in the waters of Station J, however, demonstrates that specific soluble, carbohydrate-containing substrates are unavailable to pelagic microbial communities in these polar waters. The observation that these same substrates were also not hydrolyzed in incubations carried out in other years in this same fjord (Arnosti et al., 2011; Teske et al., 2011) demonstrates that the inability to hydrolyze specific substrates is a consistent feature of these communities.

Polar microbial communities are composed of different species than those at lower latitudes (Bano et al., 2004), and exhibit enzymatic activity patterns distinct from their temperate counterparts (Arnosti et al., 2011). In order to cope with the rhythms of primary productivity and plankton succession that are unique to high-latitude environments (Hop et al., 2002), Arctic microbial communities may specialize in terms of substrate sets or controlling mechanisms for key carbon cycling processes. The observation that some soluble substrates readily hydrolyzed in lower latitude environments are only microbially available under a very specific set of circumstances supports the view that most members of Arctic pelagic microbial communities focus on a more limited range of substrates than their temperate counterparts. These substrates are hydrolyzed at considerable rates (**Figure 1**; Arnosti et al., 2011), perhaps at the expense of the ability to access a wider range of substrates, including a fraction of freshly-produced phytoplankton-derived organic matter in Svalbard waters (Thingstad et al., 2008). This streamlining of metabolic function may help explain how polar communities are able to rapidly mineralize specific organic substrates (e.g., Rich et al., 1997), even at low temperatures.

## Conflict of Interest Statement

The authors declare that the research was conducted in the absence of any commercial or financial relationships that could be construed as a potential conflict of interest.
